# Ingleby Lectures: Lecture I

**Published:** 1891-04-11

**Authors:** 


### INGLEBY LECTURES.—Lecture I.

Mr. Jordan Lloyd, after thanking the Council for the honour they had conferred on him in asking him to deliver the Ingleby lectures for the current year, said his lectures would be of the nature of a postgraduate course on many subjects of practical interest in connection with the surgical diseases of childhood; which would be dealt with only so far as he could speak of them from personal experience.

The lecturer first referred to the question of anæsthesia in children, anæsthetics being administered with greater relative frequency to children than adults. Mr. Lloyd's rule is to give an anæsthetic on every occasion when he is going to cause pain or even alarm to a child, and in his opinion fewer mistakes would be made in diagnosis if anæsthetics were always given in doubtful cases. Chloroform—pure and unadulterated—is the child's anæsthetic—occasionally to older children a mixture of four parts of chloroform and one of ether may be given. In administering an anæsthetic it is important to gain your patient's confidence. Chloroform should be given freely—the slow and nervous administrator should not be permitted to give an anæsthetic. During anæsthesia children exhibit three distinct conditions, (1) the normal, (2) the congestive, (3) the syncopal. This latter condition is important. Sometimes children have a natural tendency to faint; it calls for prompt treatment. Should alarming symptoms arise, strip all clothes off the child and hang it up by the heels. Artificial respiration may be practised in this position, and even the operation may be finished.

Collapse or Shock.—Relatively speaking the shock after an injury or operation is greater in the child than in the adult, but a good deal depends upon idiosyncracy, a trifling operation in some children causing more collapse than a severe one in others. The quicker you operate the better for the child. Most children stand loss of blood relatively as well as the adult. Shock may occur under two forms: (1) *Primary*, that immediately following an operation or injury; (2) *Secondary*, that which is seen from 12 to 48 hours afterwards. In treating shock opium is a valuable remedy even in children.

As Regards the Treatment of Wounds, Mr. Lloyd said that he had had experience of all the recognised methods of wound dressing, and that he still adhered to the view that a system of what he called a dry antiseptic, leave-well-alone dressing was the best, a system which he had practised for twelve years. He regarded sterilisation as the key-note to primary union, and the more thoroughly you can get it in your instruments, in your hands, your dressings, and in the tissues of your patient the better the results. To surgically sterilise skin surfaces (1) wash with soft soap and hot water; shave and dry; (2) rub for three or four minutes with turpentine; (3) wash again with soap and water, and cover with a cloth moistened with a 1 in 5,000 solution of perchloride of mercury. To sterilise your hands and instruments soap, hot water, a nail brush, and a little turpentine are necessary. Sponges may be looked upon as germ incubators of the most perfect construction. Squares of common lint taken fresh for each operation are safer, cheaper, and almost as convenient. Operations are best performed by the bloodless method, because not only do you save blood to your patient, but the work can be done more thoroughly and rapidly. All that is necessary is to elevate the limb and make a couple of turns round it of an ordinary piece of rubber tubing above the point where you are going to operate. It is only necessary to tie spouting vessels on the face of a wound, oozing points may be safely ignored. Mr. Lloyd drains only large wounds, and in these removes the tube altogether in the first 48 hours. Absolutely sterile wounds require little or no drainage. Before closing a wound it is a good plan to dust its surface with powdered boracic acid, which is lightly rubbed into the tissues, and which acts as a germicide against any micro-organisms which may have got shut up in the wound.

In dressing most wounds Mr. Lloyd's plan is (1) dust dry boracic acid on the edges; (2) apply a single layer of lint saturated in the glycerine of boracic acid (B. P.); (3) several thicknesses of plain absorbent wool; (4) a piece of gutta-percha tissue; (5) a soft cotton or gauze bandage. Mr. Lloyd finds as a result of this method of dressing wounds that primary union is almost the invariable rule after operation, suppuration is extremely rare, and that diseases such as pyæmia, septicæmia, and erysipelas are virtually unknown.

